# Genetic Testing at Diagnosis Has Prognostic Value in Patients with Chronic Lymphocytic Leukemia including at Early Stages

**DOI:** 10.3390/diagnostics12081802

**Published:** 2022-07-25

**Authors:** Alexia Suárez-Cabrera, Dolly Viviana Fiallo-Suárez, Ruth Stuckey, Marta Luna Uroz-de la Iglesia, Yanira Florido, Angelina Lemes-Castellano, Miguel Ángel Perera-Álvarez, Hugo Luzardo-Henríquez, Haridian de la Nuez, Paula Fernández-Caldas, Silvia de la Iglesia, María Teresa Gómez-Casares, Cristina Bilbao-Sieyro

**Affiliations:** 1 Hematology Department, Hospital Universitario de Gran Canaria Dr. Negrín, 35019 Las Palmas, Spain; asuacab@gobiernodecanarias.org (A.S.-C.); dollyfiallo@gmail.com (D.V.F.-S.); rstuckey@fciisc.es (R.S.); marta.uroz101@alu.ulpgc.es (M.L.U.-d.l.I.); floryyana@hotmail.com (Y.F.); alemcas@gobiernodecanarias.org (A.L.-C.); miguel.perera.alvarez@gmail.com (M.Á.P.-Á.); hluzhen@gobiernodecanarias.org (H.L.-H.); hnuemel@gobiernodecanarias.org (H.d.l.N.); pauliecaldas@gmail.com (P.F.-C.); siglini@gobiernodecanarias.org (S.d.l.I.); mgomcasf@gobiernodecanarias.org (M.T.G.-C.); 2 Department of Medical Sciences, Universidad de Las Palmas de Gran Canaria, 35001 Las Palmas, Spain; 3 Morphology Department, Universidad de Las Palmas de Gran Canaria, 35001 Las Palmas, Spain

**Keywords:** prognosis, chronic lymphocytic leukemia, biomarkers, patient outcome, molecular diagnostics

## Abstract

Chronic lymphocytic leukemia (CLL) has a variable clinical evolution, with some patients living treatment-free for decades while others require therapy shortly after diagnosis. In a consecutive series of 217 CLL patients, molecular biomarkers with prognostic value (IGHV status, *TP53* mutations, and cytogenetics), whose analysis is recommended prior to treatment start, were studied at diagnosis. Multivariate analyses identified prognostic variables for overall survival (OS) and time to first treatment (TTFT) and validated the CLL-IPI and IPS-E variables for all or early-stage patients (Rai 0–2/Binet A), respectively. Unmutated IGHV was associated with shorter OS and TTFT, even for early-stage patients. Lymphocyte count was not statistically significant for TTFT of early-stage patients in multivariate analysis. Our results validate the prognostic value of IGHV mutational status at diagnosis for OS and TTFT, including for early stages. Our findings suggest a role for molecular and mutational analysis at diagnosis in future prospective studies.

## 1. Introduction

Chronic lymphocytic leukemia (CLL), a lymphoproliferative malignancy of B cells, is the most frequent leukemia in Western countries with an incidence of 5/100,000 inhabitants/year [1].

CLL typically affects elderly individuals, with a median age at diagnosis of 68–70 years [2] but has a highly variable clinical course. Some patients develop an aggressive disease that requires treatment soon after diagnosis and quickly becomes refractory with a median survival of 2–3 years; whereas, other patients exhibit an indolent course, in which the disease does not progress, and have a life expectancy similar to that of the normal population [2].

The Rai and Binet clinical staging systems, developed around 40 years ago to guide prognosis, clinical practice and trials [3,4], are based on physical and laboratory examination, without requiring imaging techniques. CLL is rather unique amongst cancers since most patients are asymptomatic at diagnosis and belong to an early stage, not requiring immediate treatment. Although the Rai and Binet staging systems provide information regarding tumor burden and patient prognosis, they do not identify patients with aggressive behavior, especially in early stages, or the possible response to a certain treatment [5,6].

The origin of this heterogeneity is likely the result of the complex interaction between various intrinsic and extrinsic factors in B cells. For example, the mutational status of the variable region of immunoglobulin heavy chains (IGHV) reflects the differentiation state of the cell from which the neoplastic clone is derived and is a molecular marker with a well-established prognostic value in CLL. Patients with somatic IGHV mutations (IGHV-M or M-CLL), that is, with <98% homology with respect to the germline, have a better prognosis than patients whose B cells express a non-mutated IGHV mutational status (U-IGHV or U-CLL) [7,8]. Importantly, status of the IGHV genes also influences clinical response to chemoimmunotherapy regimens, thus, IGHV-M, especially when combined with additional prognostic factors, such as favorable cytogenetics, characterizes a CLL patient subgroup with excellent outcome following chemoimmunotherapy with fludarabine, cyclophosphamide, and rituximab [9,10,11].

Other intrinsic factors include chromosomal alterations, such as deletion of the short arms of chromosome 17 (del(17p)), chromosome 11 (del(11q), and chromosome 13 (del(13q)), or trisomy of chromosome 12, detected in approximately 80% of CLL patients [12]. Deletion of 17p, where the *TP53* suppressor gene is located, is observed in 5–8% of patients, and is associated with a marked resistance to chemotherapy [13]. Although most *TP53* mutations are accompanied by del(17p), the deletion of *TP53* in isolation is also associated with worse survival and poor response to treatment [14]. *TP53* mutations are also associated with increased genomic complexity in CLL, suggesting that TP53 malfunction promotes the development of a mutator phenotype [15].

In addition to IGHV-unmutated and del(17p) and/or *TP53* mutations, an age of over 65 years and high β_2_-microglobulin serum levels were determined to be factors of poor prognosis and found to add prognostic information to the Rai and Binet systems [16,17,18]. As a result, these variables were incorporated into predictive scores, such as the 2016 CLL International Prognostic Index (CLL-IPI) [18], now available as an online calculator (https://qxmd.com/calculate/calculator_375/cll-ipi accessed 8 July 2021), which is recognized as the most relevant current prognostic score for patients treated with chemo(immune)therapy. However, the CLL-IPI was developed as a tool to evaluate overall survival for all clinical stages of disease, while patients with asymptomatic early-stage CLL have a highly variable clinical course. As a result of this, the management of patients with early-stage CLL is challenging. Therefore, it is important to identify those patients that will progress and meet criteria for treatment initiation, for which a better insight of time to first treatment (TTFT) prediction is critical. Recently, more specific prognostic models have been developed to predict the progression and treatment-free survival in patients with asymptomatic early-stage CLL, including the IPS-E score [19,20].

The study’s objective was to analyze the clinical, biochemical (β_2_-microglobulin values), cytogenetic (deletion of 17p, 11q, 13q and trisomy 12 by FISH) and molecular (*TP53* and IGHV mutational state) characteristics at diagnosis of a series of 217 patients diagnosed with CLL at our center and determine their impact on prognosis.

## 2. Materials & Methods

### 2.1. Ethics

This retrospective non-interventional study was approved by our institutional review board (Comité Ético de Investigación Clínica, approval no. ref. 170147) on 30 November 2017 and conducted in accordance with the Declaration of Helsinki. All patient data was dissociated and anonymized; informed consent was not required due to the retrospective nature of the study and because the results did not affect the clinical management of patients.

### 2.2. Patients

A total of 217 consecutive adult CLL patients were diagnosed at our center between 2008 and 2018 (patient characteristics in Supplementary Table S1). Patients were diagnosed and treated according to the relevant International Workshop on Chronic Lymphocytic Leukemia (iwCLL) guidelines [5,6].

Clinical (lymphocyte count, nodal involvement), biochemical (β_2_-microglobulin values), cytogenetic (deletion of 17p, 11q, 13q and trisomy 12 by FISH) and molecular (*TP53* and IGHV mutational state) data was obtained from the patients’ medical records. A positive FISH result was considered for >5% of metaphases. *TP53* mutations were analyzed by Sanger sequencing (exons 4–10) and IGHV was considered unmutated at ≥98% homology, determined using IMGT/V-QUEST (www.imgt.org (accessed on 12 July 2021)) according to the ERIC-recommended protocol [21,22].

### 2.3. Statistical Methods

The relationship among categorical variables was analyzed by the Chi-squared test. Survival curves were prepared according to the Kaplan–Meier method and compared using the Log–rank test. Multivariate survival analysis was carried out using the Cox proportional hazard model for patients with available data points; only variables with a *p* ≤ 0.1 in the univariate analysis were included. Multivariate analysis was also used to validate the prognostic value of CLL-IPI variables for all patients and the IPS-E for TTFT of patients with early stage CLL [19]. For each statistical test, only patients with all available relevant data points were included. All analyses were two-sided, and statistical significance was set at a *p*-value < 0.05. Analyses were carried out using SPSS (version 22.0).

## 3. Results

### 3.1. Characteristics of the Study Population

The study series consisted of 217 patients, of which 123 (57.1%) were men and 94 (42.9%) women. The median age at diagnosis was 70 years [36–91 years]. With a median follow-up of five years (IQR: 2.6–7.7 years), 160 patients did not require therapy at any time in their evolution while 55 received treatment. Six autoimmune anemia events and four cases of autoimmune thrombopenia were recorded. In terms of survival, 22 patients (10.1%) died during follow-up, 177 (81.6%) are still alive, and follow-up was lost in 18 cases (8.3%).

At diagnosis, 154 (70.6%) were in Rai stage 0, 38 (17.4%) in stage 1, 10 (4.6%) in stage 2, 6 (2.3%) in stage 3 and 9 (4.1%) in stage 4; 174 (80.2%) were diagnosed with Binet stage A, 34 (15.7%) stage B and 9 (4.12%) stage C. The CLL-IPI could be calculated for 70 patients with all the necessary data, of whom 37 (52.9%) were low risk, 19 (27.1%) intermediate risk, 12 (17.1%) high risk and 2 (2.9%) very high risk.

### 3.2. Molecular Variables

The IGHV mutational status of 109 patients was obtained at diagnosis, of which 41 (37.6%) were non-mutated and 68 (62.4%) were mutated, similar to what has been described in the literature [7,8]. In terms of chromosomal alterations determined by FISH, del(13q) was present in 75/149 patients (50.3%), trisomy 12 in 31/149 patients (20.8%), del(11q) in 8/148 (5.4%) and del(17p) in 9/150 (6%).

### 3.3. Overall Survival (OS)

**Figure 1:**
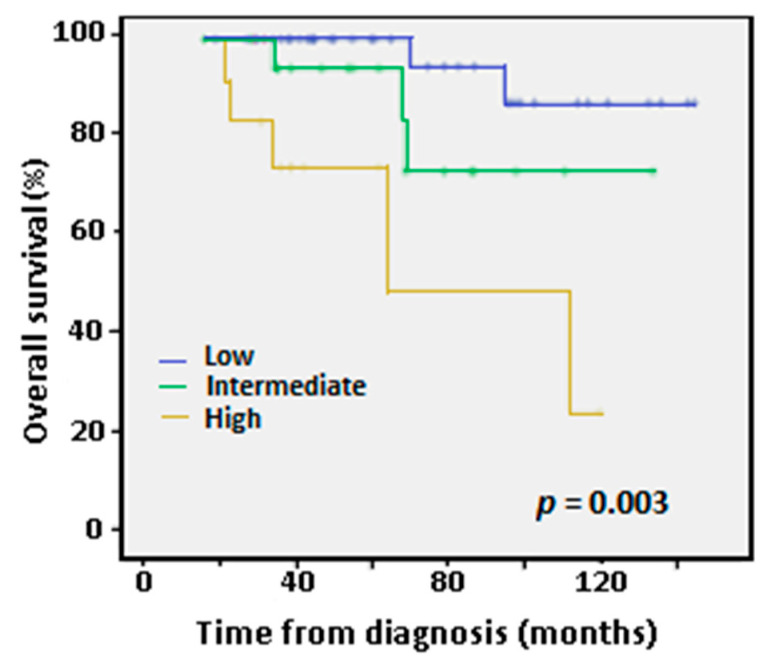
Kaplan-Meier curves for overall survival (OS) at five years according to International Prognostic Index (IPI) low-, intermediate- and high-risk categories. Only two patients belonged to the very high-risk group and were not considered in this analysis because they were of recent diagnosis and lacked follow-up. *p*-value according to Log-Rank test.

After 24 months of follow-up, 92.6% of patients were alive in the low/intermediate CLL-IPI subgroup compared to 76.9% in the high CLL-IPI subgroup, while at 72 months the percentages were 83.4% vs. 0%, respectively. Overall survival (OS) at five years for the low, intermediate and high CLL-IPI groups were clearly differentiated in Kaplan-Meier survival curves (Figure 1). OS results from our series were similar to those published by the iwCLL [6], albeit better for the intermediate and high-risk patients in our series (Supplementary Table S2). Thus, our results confirm the prognostic value of the CLL-IPI index for overall survival (*p* = 0.003).

**Figure 2:**
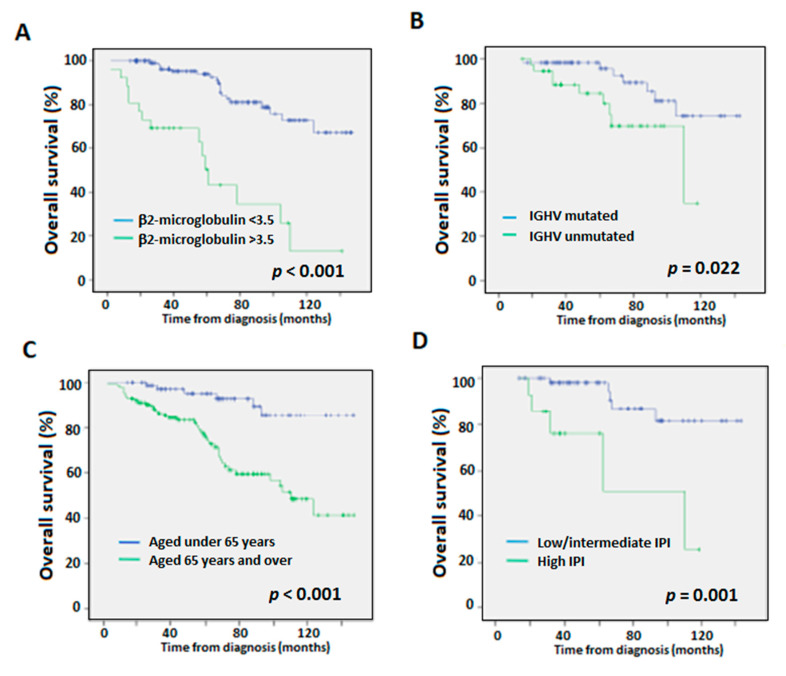
Overall survival (OS) Kaplan-Meier curves according to (**A**) β_2_-microglobulin levels, (**B**) IGHV mutational status, (**C**) age, and (**D**) low/intermediate- vs. high-risk CLL-IPI categories. *p*-values according to Log-Rank test.

With regards to the variables included in the CLL-IPI (IGHV-unmutated, del(17p) and/or *TP53* mutations, age over 65 years and high β_2_-microglobulin serum levels), high β_2_-microglobulin levels (>3.5 mg/L), IGHV-unmutated status, and age over 65 years were associated with worse OS in the univariate analyses (Figure 2). None of the chromosome alterations impacted OS. The high-risk IPI group was also an independent variable associated with worse OS compared to the low/intermediate risk groups.

In multivariate analysis, IGHV-unmutated status (41/109 patients analyzed) and Rai advanced stage (15/217 patients) were statistically associated with worse OS, while the influence of age > 65 years (*n* = 137) on OS was only marginal (Table 1).

### 3.4. Time to First Treatment (TTFT)

Comparing the low/intermediate versus the high CLL-IPI risk group, treatment-free survival was 92.6% versus 76.9% after two years, and 83.4% versus 0% after four years (*p* < 0.001).

**Figure 3:**
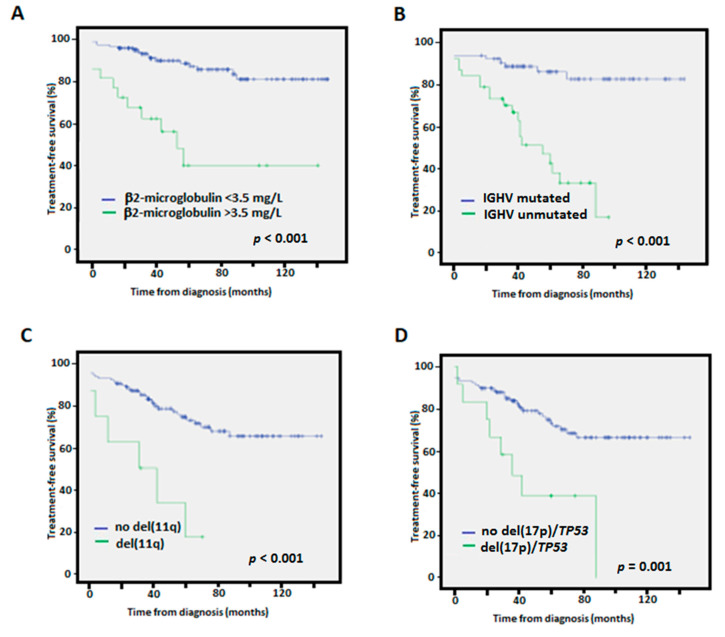
Kaplan-Meier treatment-free survival curves according to (**A**) β_2_-microglobulin levels, (**B**) IGHV mutational status, (**C**) presence of 11q deletions, (**D**) presence of 17p deletions/*TP53* mutation.

In the univariate analyses, high β_2_-microglobulin, IGHV-unmutated, del(11q), and del(17p)/*TP53* mutation were indicators of a shorter TTFT as previously described (Figure 3) [23]. High CLL-IPI also predicted worse outcome (*p* = 0.003).

In multivariate treatment free survival analyses, only IGHV-unmutated (and marginally high stage) remained as independent predictors of shorter TTFT (Table 1). The variables 11q deletions and 17p/*TP53* alterations were not considered for this analysis due to their low incidence (8/148 and 9/150 patients analyzed, respectively). Serum levels of β_2_-microglobulin lost independent value in the multivariate analysis in accordance with a previously published study [23].

### 3.5. Associations between Molecular Variables and Clinical Staging

The associations between the Rai/Binet stages and six prognostic variables—β_2_-microglobulin values, trisomy 12, del(11q), del(17p)/*TP53* mutation, IGHV mutational status and age—were studied by multivariate analysis.

Intermediate or high Rai stages (stages 2–4, *n* = 19) were significantly associated with high levels of β_2_-microglobulin (>3.5 mg/L, *p* < 0.001) and marginally associated with IGHV-unmutated status (*p* = 0.071; Table 2). Intermediate or high Binet (stages B and C, *n* = 43) were significantly associated with trisomy 12 (*p* = 0.005), IGHV-unmutated (*p* = 0.01) and marginally associated with 11q deletions (*p* = 0.077) and an age of >65 years (*p* = 0.069). Moreover, in multivariate analysis, β_2_-microglobulin was significantly associated with IGHV-unmutated (*p* = 0.002, Pearson’s χ^2^) and advanced age (*p*= 0.024, Pearson’s χ^2^).

**Figure 4:**
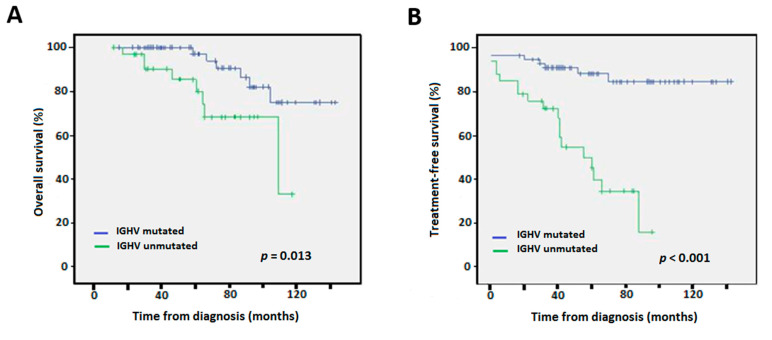
Kaplan-Meier survival curves according to IGHV mutational status for Rai stages 0–2 for (**A**) overall survival, and (**B**) time to first treatment.

The independent prognostic value of mutated IGHV maintained statistical significance when only the early stages Rai 0–2 (*n* = 206) and Binet A (*n* = 174) were considered for both OS (*p* = 0.01/0.009) and TTFT in uni- and multi-variate analyses (*p* = 0.001/0.001, respectively) (Figure 4, Table 1).

### 3.6. Validation of the IPS-E Prognostic Score

IGHV unmutated status, palpable lymph nodes and lymphocytes ≥ 15 × 10^9^/L are proposed as prognostic variables for early stage CLL patients at high risk of requiring therapy, according to the IPS-E score [19]. Multivariate analysis confirmed IGHV unmutated and palpable lymph nodes as predictive for TTFT of Rai 0–2 (HR 8.05, *p* = 0.002; HR 6.61, *p* = 0.002, respectively) and Binet A patients (HR 8.05, *p* = 0.013; HR 4.85, *p* = 0.028, respectively). Although lymphocyte count ≥ 15 × 10^9^/L was a predictor of shorter TTFT for early-stage patients in our series (Supplementary Figure S1), in multivariate analysis, it was not a statistically significant independent variable for TTFT of either Rai 0–2 or Binet A patients (Table 3).

## 4. Discussion

Our study confirmed that the CLL-IPI variables of an age of > 65 years, advanced Rai stage and IGHV-unmutated, but not β_2_-microglobulin value, had an independent impact on worse overall survival [18]. The lack of independent value of β_2_-microglobulin may be due to the series size limitation (CLL-IPI was calculated for a total of 70 patients). The lack of significance for 17p deletions/*TP53* mutations and del(11q) was probably caused by the low incidence of these alterations in our series (detected in 9/150, 6.0%, and 8/148, 5.4%, of the patients studied, respectively) and the benignity of the disease in terms of overall survival; therefore, a larger series would be required to confirm their prognostic implication. Moreover, *TP53* mutations were determined by Sanger sequencing in the majority of patients, so perhaps more mutations would have been detected if the more sensitive technique of next-generation sequencing was employed.

Our key result was that IGHV-unmutated was an independent predictor for TTFT, confirming recent publications of scores for early-stage CLL [18,19]. However, one study limitation is that only 107 of the 214 patients had IGHV mutational status determined at diagnosis. Only some clinicians requested this analysis as the determination at diagnosis is not obligatory. Thus, due to its retrospective nature, there may have been some degree of selection bias in terms of which patients were tested. Moreover, some patients lacked complete data for all variables.

Different to the IPS-E score, lymphocyte count was not predictive for TTFT of early-stage patients. Our results also confirmed the prognostic value of the high CLL-IPI risk group, which was associated with shorter TTFT versus the low/intermediate risk groups. Interestingly, the intermediate and high-risk patients in our series had better OS rates at five years than the iwCLL series [6].

In addition, we observed a significant relationship between IGHV status and intermediate-high Rai/Binet stages versus low stages, and between high β_2_-microglobulin levels and IGHV status. There is not much information about the latter relationship in the literature although it has previously been described in Binet A stages [24].

The cost of the analysis of IGHV mutational state—by multiplex PCR and several Sanger sequencing reactions—is more than justified by its prognostic value at diagnosis for OS and TTFT for all CLL patients, including those in early stages. Such genetic testing is of great importance to both patients and clinicians to address the challenge of the early identification of patients who will need a closer follow up due to shorter TTFT. Importantly, it is known that the IGHV status does not change overtime [25].

In daily practice, current guidelines recommend not treating asymptomatic patients until they present active disease, irrespective of biomarkers present at diagnosis [6,26,27]. According to the most recent consensus guidelines, the iwCLL and the National Comprehensive Cancer Network (NCCN) only recommend IGHV mutational status testing before treatment and do not explicitly recommend it at diagnosis [6,26]. Similarly, the European Society of Medical Oncology (ESMO) does not recommend the routine evaluation of IGHV status in early and asymptomatic stages and only recommends its analysis at diagnosis if the patient requests an evaluation of his or her prognostic score [27]. Nevertheless, IGHV determination at diagnosis could be of clinical interest in the context of randomized trials. As IGHV testing is already widely carried out as part of routine clinical practice for CLL patients, its application at diagnosis could be easily incorporated.

In conclusion, our findings suggest a role for molecular and mutational analysis at diagnosis in future prospective studies.

## Figures and Tables

**Table 1:** Multivariate analysis of overall survival (OS) and time to first treatment (TTFT) for the whole series and the early stages (Rai 0–2 and Binet A). *n*: number of data considered. Significant values (according to the Cox regression) are shown in bold.

	Variable		OS			TTFT		
		*n*	*p*-Value	HR	95% CI	*p*-Value	HR	95% CI
Whole series	β_2_-microglobulin (mg/L)	167	0.729	0.73	0.12–4.30	0.421	1.75	0.45–6.89
	IGHV unmutated	109	**0.01**	6.16	1.56–24.37	**0.001**	13.58	2.91–63.29
	Age > 65 years	217	0.072	3.57	0.89–14.31			
	Rai 3–4	217	**0.033**	27.22	1.30–572.00	0.081	9.67	0.76–123.70
Rai 0–2	β_2_-microglobulin (mg/L)	157	0.72	0.72	0.12–4.26	0.42	1.75	0.45–6.89
	IGHV unmutated	96	**0.01**	6.16	1.56–24.41	**0.001**	13.58	2.91–63.29
	Age > 65 years	204	0.073	3.57	0.89–14.32			
Binet A	β_2_-microglobulin (mg/L)	137	0.912	1.069	0.24–5.03	0.219	2.19	0.63–7.62
	IGHV unmutated	77	**0.009**	5.96	1.56–22.72	**0.001**	14.08	3.04–65.28
	Age > 65 years	176	0.066	3.59	0.92–14.01			

**Table 2:** Association of the five CLL-IPI prognostic variables [18] and del(11q) with Rai/Binet staging. *n*: number of data considered. Significant values (according to the Cox regression) are shown in bold.

Variable		*n*	N (%)		*p*-Value	N (%)		*p*-Value
			Rai 0–2	Rai 3–4		Binet A	Binet B-C	
β_2_-microglobulin (mg/L)	≤3.5	150	130 (95.6)	6 (4.4)	**<0.001**	117 (8.4)	20 (14.6)	0.147
	>3.5		20 (74.1)	7 (25.9)		20 (74.1)	7(25.9)	
Trisomy 12	No	123	101 (91)	10 (9)	0.155	90 (81.1)	21 (18.9)	**0.005**
	Yes		22 (81.5)	5 (18.5)		15 (55.6)	12 (44.4)	
Del(11q)	No	122	116 (89.9)	13 (10.1)	0.19	100 (77.5)	29 (22.5)	0.077
	Yes		6 (75)	2 (25)		4 (50)	4 (50)	
Del(17p)/*TP53* mutation	No	123	112 (88.9)	14 (11.1)	0.768	96 (76.2)	30 (23.8)	0.926
	Yes		11 (91.7)	1 (8.3)		9 (75)	3 (25)	
IGHV mutated	No	90	58 (93.5)	4(6.5)	0.071	53 (84.1)	10 (15.9)	**0.01**
	Yes		32 (82.1)	7 (17.9)		24 (61.5)	15 (38.5)	
Age	≤65 years	192	73 (91.3)	7 (8.8)	0.397	59 (73.8)	21 (26.3)	0.069

**Table 3:** Multivariate analysis of variables for TTFT of early stage CLL patients according to the IPS-E score [19]. Rai stages 0–2 and Binet stage A were considered as early stages. Significant values (according to the Cox regression) are shown in bold.

	Rai 0–2				Binet A			
Variable	*n*	HR	*p*-Value	95% CI	*n*	HR	*p*-Value	95% CI
Lymphocytes ≥ 15 × 10^9^/L	79	1.62	0.384	0.55–4.84	67	1.85	0.40	0.45–7.80
IGHV unmutated	79	8.05	**0.002**	2.10–30.87	67	8.05	**0.013**	1.54–42.10
Nodal involvement	79	6.61	**0.002**	2.06–21.52	67	4.85	**0.028**	1.18–19.92

*n*: number of data considered; 95% CI: 95% confidence interval.

## Data Availability

The datasets generated and analysed for this study are available from the corresponding author upon reasonable request.

## Supplementary Materials

The following supporting information can be downloaded at: https://www.mdpi.com/article/10.3390/diagnostics12081802/s1, Figure S1: Kaplan-Meier treatment-free survival curves for early-stage patients according to lymphocyte count, (A) Rai stages 0–2, and (B) Binet stages A; Table S1: Characteristics of the consecutive series of adult CLL patients; Table S2: Five-year overall survival of the CLL-IPI categories versus our series of CLL patients.

## References

[1] Sant M., Allemani C., Tereanu C., De Angelis R., Capocaccia R., Visser O., Marcos-Gragera R., Maynadié M., Simonetti A., Lutz J.-M. (2010). Incidence of hematologic malignancies in Europe by morphologic subtype: Results of the HAEMACARE project. Blood.

[2] Hallek M. (2015). Chronic lymphocytic leukemia: 2015 Update on diagnosis, risk stratification, and treatment. Am. J. Hematol..

[3] Rai K.R., Sawitsky A., Cronkite E.P., Chanana A.D., Levy R.N., Pasternack B.S. (1975). Clinical staging of chronic lymphocytic leukemia. Blood.

[4] Binet J.L., Auquier A., Dighiero G., Chastang C., Piguet H., Goasguen J., Vaugier G., Potron G., Colona P., Oberling F. (1981). A new prognostic classification of chronic lymphocytic leukemia derived from a multivariate survival analysis. Cancer.

[5] Hallek M., Cheson B.D., Catovsky D., Caligaris-Cappio F., Dighiero G., Döhner H., Hillmen P., Keating M.J., Montserrat E., Rai K.R. (2008). Guidelines for the diagnosis and treatment of chronic lymphocytic leukemia: A report from the International Workshop on Chronic Lymphocytic Leukemia updating the National Cancer Institute–Working Group 1996 guidelines. Blood.

[6] Hallek M., Cheson B.D., Catovsky D., Caligaris-Cappio F., Dighiero G., Döhner H., Hillmen P., Keating M., Montserrat E., Chiorazzi N. (2018). iwCLL guidelines for diagnosis, indications for treatment, response assessment, and supportive management of CLL. Blood.

[7] Hamblin T.J., Davis Z., Gardiner A., Oscier D.G., Stevenson F. (1999). Unmutated Ig V(H) genes are associated with a more aggressive form of chronic lymphocytic leukemia. Blood.

[8] Damle R.N., Wasil T., Fais F., Ghiotto F., Valetto A., Allen S.L., Buchbinder A., Budman D., Dittmar K., Kolitz J. (1999). Ig V gene mutation status and CD38 expression as novel prognostic indicators in chronic lymphocytic leukemia. Blood.

[9] Thompson P.A., Tam C.S., O’Brien S.M., Wierda W.G., Stingo F.C., Plunkett W., Smith S.C., Kantarjian H.M., Freireich E.J., Keating M.J. (2016). Fludarabine, cyclophosphamide, and rituximab treatment achieves long-term disease-free survival in IGHV-mutated chronic lymphocytic leukemia. Blood.

[10] Rossi D., Terzi-Di-Bergamo L., De Paoli L., Cerri M., Ghilardi G., Chiarenza A., Bulian P., Visco C., Mauro F.R., Morabito F. (2015). Molecular prediction of durable remission after first-line fludarabine-cyclophosphamide-rituximab in chronic lymphocytic leukemia. Blood.

[11] Fischer K., Bahlo J., Fink A.M., Goede V., Herling C.D., Cramer P., Langerbeins P., von Tresckow J., Engelke A., Maurer C. (2016). Long-term remissions after FCR chemoimmunotherapy in previously untreated patients with CLL: Updated results of the CLL8 trial. Blood.

[12] Döhner H., Stilgenbauer S., Benner A., Leupolt E., Kröber A., Bullinger L., Döhner K., Bentz M., Lichter P. (2000). Genomic Aberrations and Survival in Chronic Lymphocytic Leukemia. N. Engl. J. Med..

[13] Campo E., Cymbalista F., Ghia P., Jäger U., Pospisilova S., Rosenquist R., Schuh A., Stilgenbauer S. (2018). *TP53* aberrations in chronic lymphocytic leukemia: An overview of the clinical implications of improved diagnostics. Haematologica.

[14] Zenz T., Vollmer D., Trbusek M., Smardova J., Benner A., Soussi T., Helfrich H., Heuberger M., Hoth P., Fuge M. (2010). TP53 mutation profile in chronic lymphocytic leukemia: Evidence for a disease specific profile from a comprehensive analysis of 268 mutations. Leukemia.

[15] Seiffert M., Dietrich S., Jethwa A., Glimm H., Lichter P., Zenz T. (2011). Exploiting biological diversity and genomic aberrations in chronic lymphocytic leukemia. Leuk. Lymphoma.

[16] Wierda W.G., O’Brien S., Wang X., Faderl S., Ferrajoli A., Do K.-A., Cortes J., Thomas D., Garcia-Manero G., Koller C. (2007). Prognostic nomogram and index for overall survival in previously untreated patients with chronic lymphocytic leukemia. Blood.

[17] Pflug N., Bahlo J., Shanafelt T.D., Eichhorst B.F., Bergmann M.A., Elter T., Bauer K., Malchau G., Rabe K.G., Stilgenbauer S. (2014). Development of a comprehensive prognostic index for patients with chronic lymphocytic leukemia. Blood.

[18] International CLL-IPI working group (2016). An international prognostic index for patients with chronic lymphocytic leukaemia (CLL-IPI): A meta-analysis of individual patient data. Lancet Oncol..

[19] Condoluci A., Di Bergamo L.T., Langerbeins P., Hoechstetter M.A., Herling C.D., De Paoli L., Delgado J., Rabe K.G., Gentile M., Doubek M. (2020). International prognostic score for asymptomatic early-stage chronic lymphocytic leukemia. Blood.

[20] Cohen J.A., Rossi F.M., Zucchetto A., Bomben R., Terzi-Di-Bergamo L., Rabe K.G., Degan M., Steffan A., Polesel J., Santinelli E. (2019). A laboratory-based scoring system predicts early treatment in Rai 0 chronic lymphocytic leukemia. Haematologica.

[21] Malcikova J., Tausch E., Rossi D., Sutton L.A., Soussi T., Zenz T., Kater A.P., Niemann C.U., Gonzalez D., Davi F. (2018). ERIC recommendations for TP53 mutation analysis in chronic lymphocytic leukemia—Update on methodological approaches and results interpretation. Leukemia.

[22] Rosenquist R., Ghia P., Hadzidimitriou A., Sutton L.-A., Agathangelidis A., Baliakas P., Darzentas N., Giudicelli V., Lefranc M.-P., Langerak A.W. (2017). Immunoglobulin gene sequence analysis in chronic lymphocytic leukemia: Updated ERIC recommendations. Leukemia.

[23] Wierda W.G., O’Brien S., Wang X., Faderl S., Ferrajoli A., Do K.-A., Garcia-Manero G., Cortes J., Thomas D., Koller C.A. (2011). Multivariable Model for Time to First Treatment in Patients With Chronic Lymphocytic Leukemia. J. Clin. Oncol..

[24] Gentile M., Cutrona G., Neri A., Molica S., Ferrarini M., Morabito F. (2009). Predictive value of beta2-microglobulin (beta2-m) levels in chronic lymphocytic leukemia since Binet A stages. Haematologica.

[25] Chiorazzi N., Rai K.R., Ferrarini M. (2005). Chronic lymphocytic leukemia. N. Engl. J. Med..

[26] NCCN Guidelines Version 3.2022. Chronic Lymphocytic Leukemia/Small Lymphocytic Lymphoma. https://www.nccn.org/guidelines.

[27] Eichhorst B., Robak T., Montserrat E., Ghia P., Niemann C., Kater A., Gregor M., Cymbalista F., Buske C., Hillmen P. (2020). Chronic lymphocytic leukaemia: ESMO Clinical Practice Guidelines for diagnosis, treatment and follow-up. Ann. Oncol..

