# Supplementary material for: Genetic Testing at Diagnosis Has Prognostic Value in Patients with Chronic Lymphocytic Leukemia including at Early Stages
Source: Diagnostics (Basel). 2022 Jul 25;12(8):1802. doi: 10.3390/diagnostics12081802 (PMC9394282; doi:10.3390/diagnostics12081802)
Supplement: Supplementary file 1 [file diagnostics-12-01802-s001.zip › diagnostics-1792825-supplementary.pdf]

diagnostics

MDPI

SUPPLEMENTARY DATA

Supplementary Figure S1. Kaplan-Meier treatment-free survival curves for early-stage patients according to lymphocyte count, **A)** Rai stages 0-2, and **B)** Binet stages A.

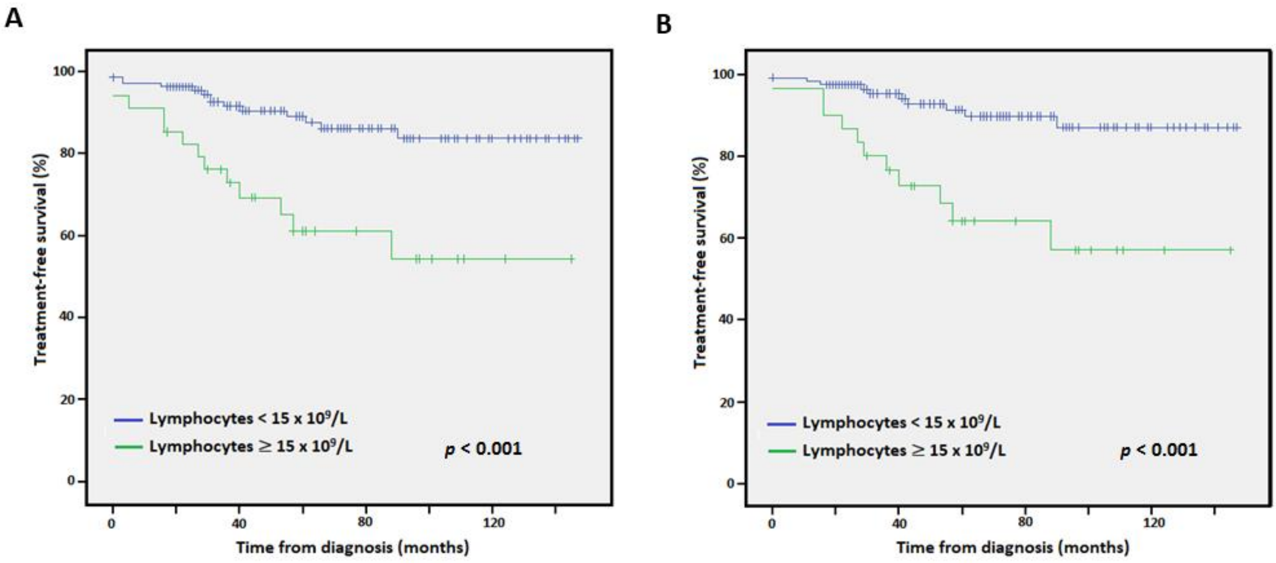

Supplementary Table S1. Characteristics of the consecutive series of adult CLL patients.

Characteristic	Total	*n*/mean (% or range)
**Age, mean (years)**	217	70 (16-91)
**Sex**	217	
Male		123 (57.1)
Female		94 (42.9)
**Rai stage**	217	
0		154 (70.6)
I		38 (17.4)
II		10 (4.6)
III-IV		15 (6.9)
**Binet stage**	217	
A		174 (80.2)
B		34 (15.7)
C		9 (4.1)
**IPI**	70	
Low		37 (52.9)
Intermediate		19 (27.1)
High		12 (17.1)
Very high		2 (2.9)
**Treatment**	215	
No		160 (74.4)
Yes		55 (25.6)
**Treatment type**	57	
FCR		15
Chlorambucil		15
Ibrutinib		8
R-Benda		7
Chlorambucil + Obinutuzumab		5
Rituximab		3
CHOP/R-CHOP		2
Alentuzumab + TPH		1
Radiotherapy		1
**Beta-2 microglobulin, mean (mg/L)**	150	2.4 (1.1-16.1)
**Hierarchic FISH category**		
17p deletion	150	9 (6)
11q deletion	148	8 (5.4)
12 trisomy	149	31 (20.8)
13q deletion	149	75 (50.3)
**IGHV mutation status**	109	
Unmutated		41 (37.6)
Mutated		68 (62.4)
**Exitus**	217	
No		177 (81.6)
Yes		22 (10.1)
Lost to follow-up		18 (8.3)

Supplementary Table S2. Five-year overall survival of the CLL-IPI categories [18] versus our series of CLL patients. OS, overall survival; iwCLL, International Workshop on Chronic Lymphocytic Leukemia (2018) [6].

			**iwCLL series**			**Our series**
**CLL-IPI category**		***n***	**5-year OS (%)**		***n***	**5-year OS (%)**
Low-risk		341	93.2		36	100.0
Intermediate-risk		474	79.3		19	94.0
High-risk		337	63.3		12	74.0
Very high-risk		62	23.3		2	-
